# Association of Blood Pressure Within 6 h After Endovascular Thrombectomy and Functional Outcomes in Ischemic Stroke Patients With Successful Recanalization

**DOI:** 10.3389/fneur.2022.860124

**Published:** 2022-04-14

**Authors:** Xuening Zhang, Ting Cui, Qiange Zhu, Changyi Wang, Anmo Wang, Yuan Yang, Shucheng Li, Fayun Hu, Bo Wu

**Affiliations:** ^1^Department of Neurology, West China Hospital, Sichuan University, Chengdu, China; ^2^Second Department of Neurology, Shaanxi Provincial People's Hospital, Xi'an, China; ^3^Department of Rehabilitation Medicine Center, West China Hospital, Sichuan University, Chengdu, China; ^4^Key Laboratory of Rehabilitation Medicine in Sichuan Province, West China Hospital, Sichuan University, Chengdu, China

**Keywords:** brain ischemia, cerebral Infarction, stroke, endovascular procedures, thrombectomy, cerebral revascularization, blood pressure, hypertension

## Abstract

**Background and Purpose:**

Blood pressure in the days following endovascular thrombectomy (EVT) can influence functional outcomes of patients who have suffered an acute ischemic stroke, but whether the same is true of blood pressure during the first few hours after EVT is unclear.

**Methods:**

Several blood pressure parameters were retrospectively analyzed in acute ischemic stroke patients who underwent EVT at West China Hospital from March 2016 to December 2019. Baseline blood pressure, speed of blood pressure reduction, postoperative blood pressure, degree of blood pressure reduction, and quality of blood pressure management were evaluated during the first 24 h after EVT. We explored whether these parameters during different time windows correlated significantly with patients' modified Rankin Scale (mRS) score at 90 days.

**Results:**

Analysis of 163 patients showed that poor functional outcome (mRS scores 3–6) correlated significantly with higher postoperative blood pressure and worse blood pressure management during the first 6 h after EVT. Postoperative systolic blood pressure at 37 min after EVT was significantly higher in patients with poor outcome (141 mmHg) than in those with good outcome (mRS scores 0–2; 122 mmHg, *p* = 0.006), and systolic pressure >136 mmHg at this time point was associated with a significantly higher risk of poor outcome, before and after adjusting for other risk factors (adjusted OR 0.395, 95% CI 0.20–0.79).

**Conclusions:**

Among acute ischemic patients who successfully undergo recanalization, adequate blood pressure management during the first 30–40 min after EVT may be important for ensuring good 90-day functional outcomes.

## Introduction

Since its description in 2015, endovascular thrombectomy (EVT) has become a standard method for treating large-vessel acute ischemic stroke within 6 h of onset (1), and large clinical trials suggest that it remains effective even when performed up to 24 h after onset (2, 3). How blood pressure management before and after EVT affects patient outcomes remains uncertain, which makes it difficult to optimize such management.

Several studies have suggested that lower blood pressure during 24 h after EVT is associated with a better prognosis (4–6), but we are unaware of studies examining whether blood pressure sooner after EVT is also important. This is an important question, given that the blood pressure of most acute ischemic stroke patients stabilizes at 6–8 h after EVT (7).

Therefore, we performed a retrospective observational study to explore whether blood pressure parameters during the first 6 h after EVT significantly influence functional outcomes.

## Methods

### Study Population

Medical records were retrospectively examined for acute ischemic stroke patients who underwent EVT at West China Hospital from March 2016 to December 2019. Follow-up information was derived from a database we set up prospectively since 2016 for all ischemic stroke patients admitted to West China Hospital and underwent reperfusion. Patients were considered eligible for the present study if they were older than 18 years, underwent emergency EVT for acute ischemic stroke affecting the anterior circulation [including the internal carotid artery (ICA), middle cerebral artery (MCA), and anterior cerebral artery (ACA)], and underwent successful recanalization after EVT [defined as the modified Thrombolysis in Cerebral Infarction score (mTICI) ≥2b]. Patients were excluded if blood pressure data were missing for any of the following time points: on admission, every hour during the first 6 h after EVT, and every 2 h until 24 h after EVT. Patients were also excluded if computed tomography (CT) data were unavailable within 24 h after EVT, or if the modified Rankin Scale (mRS) score at 90 days after EVT was unknown.

This study was approved by the Institutional Review Board of West China Hospital, which waived the requirement for written informed consent because, at the time of treatment, patients or their legal guardians gave informed oral consent for the patient's anonymized medical data to be analyzed and published for research purposes.

### Procedures and Treatment

Patient eligibility for EVT was determined based on established clinical guidelines (8). Vasopressors or the antihypertensives urapidil hydrochloride or nicardipine hydrochloride were injected at the attending physician's discretion to control blood pressure. The blood pressure target of patients during the first 24 h after EVT in this study was 120 mmHg, which reflects the standard practice at our hospital for acute ischemic stroke patients who successfully undergo recanalization.

### Blood Pressure Parameters

BP values at set points were measured non-invasively. Based on the blood pressure information, we calculated parameters reflecting 5 aspects of blood pressure profiles: (1) baseline BP level: including admission SBP and the first postoperative SBP (FPO SBP). FPO SBP was the SBP value patients on their first measurement when they returned to the ward after EVT; (2) postoperative BP level: including mean SBP and minimum SBP; (3) degree of BP reduction: including FPO SBP-mean SBP, FPO SBP-minimum SBP (FPO SBP-mean SBP)/FPO SBP and (FPO SBP-minimum SBP)/FPO SBP; (4) velocity of BP reduction: indicated by the time from FPO SBP to the minimum SBP record during the first 6 h after EVT; (5) BP management quality: including maximum SBP and the time below 120 and 140 mmHg. The threshold of 140 mmHg was chosen because several studies have proposed it as a target blood pressure during the first 24 h after EVT for patients with successful recanalization (9–11).

Time points of measurements were divided into intervals of 0.5 h. Measurements for the abovementioned parameters, excluding baseline blood pressure and speed of BP reduction, were categorized into the following four intervals after EVT: 1–6, 7–12, 13–18, and 19–24 h.

### Outcomes and Covariates

The primary study outcome was the mRS score at 90 days, which was determined through telephone interviews with patients or their legal guardians. Good outcome was defined as mRS scores 0–2, while poor outcome was defined as mRS scores 3–6. The secondary study outcome was intracerebral hemorrhage (ICH) which may have occurred during hospitalization and malignant cerebral edema (defined as the development of clinical signs of herniation, including a decrease in consciousness and/or anisocoria), accompanied by prominent imaging evidence of brain swelling (i.e., midline shift of brain structure or effacement of basal cisterns).

We collected data on numerous covariates to adjust for their potential influence in our analyses and thereby isolate the influence of blood pressure at different times after EVT. Covariates included age; sex; history of hypertension, diabetes, coronary heart disease, cardiac dysfunction, and atrial fibrillation; pre-EVT laboratory findings, such as fasting blood glucose, low-density lipoprotein, prothrombin time, and international normalized ratio; Alberta Stroke Program Early Computed Tomography Score (ASPECTS) on pre-EVT computed tomography; score on the National Institutes of Health Stroke Scale (NIHSS) at admission; occluded segment; prior treatment with intravenous alteplase; time from onset to puncture (OTP); postoperative hypotension, underwent decompressive craniectomy and postoperative residual moderate to severe stenosis.

### Statistical Analysis

Continuous data were expressed as mean ± standard deviation if normally distributed, or as median (interquartile range, IQR) otherwise. Categorical data were expressed as n (%). The normality of data distribution was assessed using the Shapiro-Wilk test. Analyses were conducted initially across all patients for whom covariate data were complete. Differences between patients with poor or good outcome and between patients with or without intracerebral hemorrhage were assessed for significance using a chi-squared test in the case of categorical data, Student's *t* test in the case of normally distributed continuous data, or the Mann-Whitney U test in the case of skewed continuous data.

Variables with a *p* < 0.1 in univariate analysis were entered into two logistic regression models: Model 1 adjusted for gender, fasting blood glucose, and NIHSS; Model 2 adjusted for age and ASPECTS. Multiple imputation was used to fill in missing values. Stratified analysis was performed based on whether systolic blood pressure at admission was <140 or ≥140 mmHg, patients had ICA occlusion or not, time from onset to thrombectomy was ≤6h, and patients had cardiac dysfunction or not. We set the systolic blood pressure threshold at 140 mmHg because a previous study found that systolic pressures higher than this value were associated with higher mRS score (9).

We also performed four analyses to examine closely the potential relationship between the first postoperative systolic blood pressure and the primary study outcome. First, we created a generalized additive model to describe how the 90-day mRS score varied with first postoperative systolic blood pressure. Second, we created a linear regression model to describe how the risk of poor functional outcome changed with increasing first postoperative systolic blood pressure. Third, we determined the first postoperative systolic blood pressure that best predicted good or poor functional outcome based on receiver operating characteristic curves and the Youden index. Fourth, we dichotomized first postoperative systolic blood pressure values at the cut-off values and assessed the association of each group of values with functional outcome using the chi-squared test and the abovementioned logistic regression models.

All regressions and imputations were conducted using SPSS for Windows (version 26.0; IBM, Chicago, IL, USA). Data were plotted and Student tests were performed using GraphPad Prism for Windows (version 8.01; GraphPad Software, San Diego, CA, USA). Generalized additive modeling was conducted using R (version 4.1). Differences were considered significant at a level of α = 0.05.

## Results

### Characteristics of the Study Population

**Figure 1:**
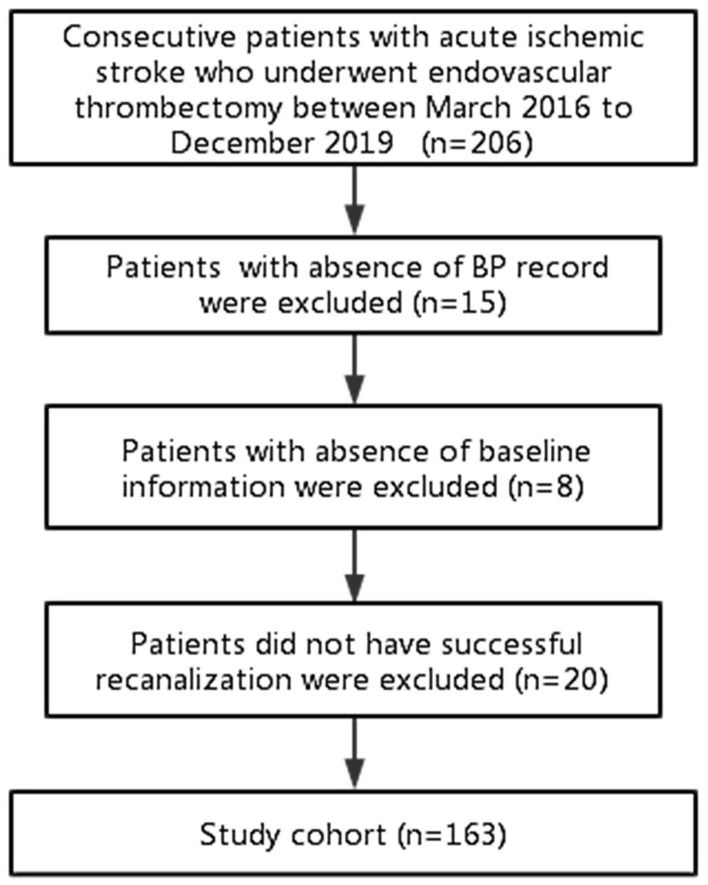
Flowchart of patient inclusion.

Of the 206 consecutive patients reviewed for enrollment, 163 were included in the final analysis (Figure 1). The median age of the 163 patients was 69 years (IQR 59–78) and mean NIHSS was 15 (SD 5) (Table 1). One-third of patients (49, 30.1%) had ICA occlusion. All patients underwent EVT under general anesthesia.

**Table 1:** Baseline characteristics of study population compared between patients with good and poor functional outcome (mRS 0–2 vs. 3–6).

	**Total number**	**90-day mRS**			**Missing (good/poor)**
		**Good, *n* (%)**	**Poor, *n* (%)**	***p*-value (unadjusted)**	
Number of patients	163	73 (44.8)	90 (55.2)		
**Patient characteristics**					
Age, y, median (IQR)	69 (59–78)	63 (53–74)	74 (64–80)	**<0.001**	
Female	74 (0.45)	25 (0.34)	49 (0.54)	**0.012**	
ASPECTS 6–10	103 (68.2)	57 (86.4)	46 (54.1)	**<0.001**	7/5
NIHSS score (mean ± SD)	15 ± 5	13 ± 6	17 ± 5	**<0.001**	1/0
Previous use of IV thrombolysis	56 (34.4)	27 (37.0)	29 (32.2)	0.638	
OTP [mean (SD)]	4.64 (1.11)	4.58 (0.99)	4.69 (1.21)	0.56	3/5
Postoperative hypotension	12 (7.4)	3 (4.1)	9 (10.0)	0.258	
Decompressive craniectomy	4 (2.5)	1 (1.4)	3 (3.4)	0.76	
Malignant cerebral edema	23 (14.1)	2 (2.7)	21 (23.3)	**<0.001**	
Postoperative residual stenosis (moderate or severe)	25 (15.6)	11 (15.5)	14 (15.7)	1	
ICH during hospitalization	61 (37.4)	20 (27.4)	41 (45.6)	**0.026**	
**Medical history**					
Hypertension	80 (49.1)	33 (45.2)	47 (52.2)	0.463	
Diabetes mellitus	32 (19.6)	10 (13.7)	22 (24.4)	0.129	
Coronary heart disease	24 (14.7)	10 (13.7)	14 (15.6)	0.912	
Cardiac dysfunction	44 (27.0)	18 (24.7)	26 (28.9)	0.669	
Atrial fibrillation	75 (46.0)	29 (39.7)	46 (51.1)	0.196	
**Laboratory index**					
Fasting blood glucose, median (IQR)	7.3 (6.3–9)	6.9 (6.1–8.3)	7.5 (6.7–9.6)	**0.012**	0/1
LDL, median (IQR)	2.4 (1.9–2.9)	2.7 (2.1–2.9)	2.4 (1.9–3)	0.201	0/1
PT, median (IQR)	11.5 (10.8–12.4)	11.4 (10.8–12.4)	11.7 (10.9–12.3)	0.597	0/2
INR, median (IQR)	1.01 (0.97–1.10)	1.02 (0.95–1.09)	1.01 (0.97–1.11)	0.344	0/2
**Occluded segment**					
ICA	49 (30.1)	18 (24.7)	31(34.4)	0.237	
M1	71 (43.6)	33 (45.2)	38 (42.2)	0.823	
M2	36 (22.1)	20 (27.4)	16 (17.8)	0.2	
Other^*^	9 (5.5)	2 (2.7)	7 (7.8)	0.291	

*mRS, modified Rankin scale; IQR, interquartile range; ASPECT, alberta stroke program early CT score; NIHSS, national institute of health stroke Scale; SD, standard deviation; IV, intravenous; OTP, time from onset to puncture; ICH, intracerebral hemorrhage; PT, prothrombin time; INR, international normalized ratio; ICA, internal carotid artery; M(segment), middle cerebral artery; A(segment), anterior cerebral artery*.

* *A1/A2/M3 occlusion. Bold values means p-values ≤ 0.05*.

Just over half of patients (90, 55.2%) had poor functional outcome at 90 days. These patients were older and showed more severe infarction reflected in a lower rate of ASPECTS > 6 and higher NIHSS score than patients with good functional outcome.

### Associations of Functional Outcome With Various Blood Pressure Parameters During the First 24 h After EVT

Patients who experienced good or poor functional outcomes did not differ significantly in systolic blood pressure at admission (141 ± 24 vs. 149 ± 27 mmHg, *p* = 0.072, Table 2). In contrast, those with poor functional outcome showed significantly higher systolic blood pressure at the first postoperative measurement (141 vs. 122 mmHg, *p* = 0.006), which was taken an average of 37 min (95%CI 34–40) after EVT.

**Table 2:** Associations between BP parameters and functional outcomes (mRS 0–2 vs. 3–6).

		**90-day mRS**				**Missing (good/poor)**
		**Total**	**Good (74)**	**Poor (109)**	***p*-value (unadjusted)**	
**Baseline BP level**						
Admission SBP (mean ± SD)		145 ± 26	141 ± 24	149 ± 27	0.072	0/5
FPO SBP [median (IQR)]		133 (112–152)	122 (107–148)	141 (117–155)	**0.006**	
**Postoperative BP level**						
Mean SBP (mean ± SD)	A	128 ± 14	125 ± 14	130 ± 14	**0.023**	
	B	121 ± 14	120 ± 14	122 ± 13	0.212	
	C	121 ± 14	121 ± 15	122 ± 13	0.685	
	D	123 ± 15	122 ± 15	125 ± 16	0.253	
Minimum SBP (mean ± SD)	A	110 ± 13	107 ± 12	112 ± 14	**0.011**	
	B	112 ± 14	112 ± 13	113 ± 14	0.626	
	C	111 ± 14	111 ± 14	111 ± 14	0.888	
	D	114 ± 15	112 ± 15	114 ± 16	0.402	
**BP reduction degree**						
FPO SBP-Mean SBP (mean ± SD)	A	6 ± 21	3 ± 20	9 ± 21	0.055	
	B	13 ± 29	8 ± 28	17 ± 30	0.059	
	C	13 ± 29	7 ± 28	18 ± 28	**0.020**	
	D	11 ± 28	6 ± 27	15 ± 28	**0.050**	
FPO SBP-Minimum SBP (mean ± SD)	A	24 ± 25	21 ± 24	27 ± 26	0.136	
	B	22 ± 30	16 ± 28	27 ± 31	**0.028**	
	C	23 ± 29	17 ± 28	28 ± 30	**0.011**	
	D	21 ± 30	16 ± 27	26 ± 31	**0.026**	
(FPO SBP-Mean SBP)/FPO SBP (mean ± SD)	A	0.02 ± 0.15	−0.01 ± 0.15	0.04 ± 0.14	**0.041**	
	B	0.06 ± 0.20	0.03 ± 0.20	0.09 ± 0.20	0.064	
	C	0.06 ± 0.20	0.02 ± 0.21	0.10 ± 0.19	**0.017**	
	D	0.05 ± 0.19	0.02 ± 0.20	0.08 ± 0.19	**0.045**	
(FPO SBP-Minimum SBP)/FPO SBP (mean ± SD)	A	0.16 ± 0.14	0.14 ± 0.14	0.17 ± 0.14	0.155	
	B	0.13 ± 0.20	0.09 ± 0.19	0.16 ± 0.20	**0.032**	
	C	0.14 ± 0.19	0.10 ± 0.19	0.17 ± 0.19	**0.011**	
	D	0.13 ± 0.20	0.09 ± 0.19	0.16 ± 0.20	**0.030**	
**Velocity of BP reduction**						
Time from FPO to the minimum SBP A (h)		2.5 (1.0-5.0)	2.5 (0.5-4.5)	3.0 (1.1-5.0)	0.104	
**BP management quality median (mean)**						
Time under 120 mmHg (h)	A	2 (2.52)	2.5 (2.79)	2 (2.29)	0.119	
	B	3 (3.11)	3 (3.24)	3 (3.01)	0.430	
	C	3 (3.6)	3 (4.15)	3 (3.14)	0.628	
	D	2 (2.84)	4 (3.12)	2 (2.61)	0.290	
Time under 140 mmHg (h)	A	5 (4.61)	6 (4.89)	5 (4.38)	**0.025**	
	B	6 (5.33)	6 (5.45)	6 (5.23)	0.564	
	C	6 (5.39)	6 (5.37)	6 (5.41)	0.805	
	D	6 (5.15)	6 (5.18)	6 (5.12)	0.548	
Maximum SBP (mean ± SD)	A	152 ± 25	146 ± 23	157 ± 25	**0.005**	
	B	131 ± 16	129 ± 15	133 ± 16	0.099	
	C	131 ± 16	129 ± 16	132 ± 15	0.144	
	D	132 ± 16	129 ± 17	134 ± 16	**0.046**	

*mRS, modified Rankin scale; BP, blood pressure; SBP, systolic blood pressure; SD, standard deviation; FPO SBP, the first postoperative SBP; h, hours; A, 0–6 h following procedural; B, 7–12 h following procedural; C, 13–18 h following procedural; D, 19–24 h following procedural. Bold values means p-values ≤ 0.05*.

During the first 6 h after EVT, patients with poor functional outcomes showed significantly higher postoperative blood pressure, in terms of mean SBP (*p* = 0.023) and minimum SBP (*p* = 0.011) (Table 2). Those patients also showed worse quality of blood pressure management, reflected in significantly higher maximum SBP (*p* = 0.005) and shorter time when SBP was below 140 mmHg (*p* = 0.025). During the other time windows analyzed during the first 24 h after EVT, patients who experienced good or poor functional outcomes did not differ significantly in any of the blood pressure parameters except the degree of blood pressure reduction (Table 2).

Consistent with these results, logistic regression Model 1 showed significant correlations of functional outcome with the first postoperative measurement of systolic blood pressure as well as minimum and maximum values of systolic blood pressure during the first 6 h after EVT. However, the significance did not remain when adjusted for age and ASPECT in Model 2 (Supplementary Table 1).

When we dichotomized patients based on whether the first postoperative measurement of systolic blood pressure was <140 or ≥140 mmHg, we found that none of the other blood pressure parameters correlated significantly with the functional outcome in both subgroups (data not shown). Among the subgroups of patients with ICA occlusion, time from onset to puncture (<6h), or without cardiac dysfunction, good functional outcome significantly correlated with lower FPO BP, lower postoperative blood pressure, and better quality of blood pressure management during the first 6 h. Among patients without ICA occlusion, functional outcome correlated significantly with maximum and minimum systolic blood pressures during the first 6 h. Among patients with admission SBP ≥140 mmHg, good functional outcome correlated significantly with lower FPO BP and lower maximum SBP during the first 6 h (Supplementary Table 2). Our additional comparison of patients with or without ICA occlusion showed that the two subgroups did not differ significantly in systolic blood pressure at admission or the first postoperative measurement, nor in mean or maximum systolic blood pressures during any time windows within the first 24 h after EVT (data not shown).

### Further Analysis of the Association Between Functional Outcome and the First Postoperative Measurement of Systolic Blood Pressure

**Figure 2:**
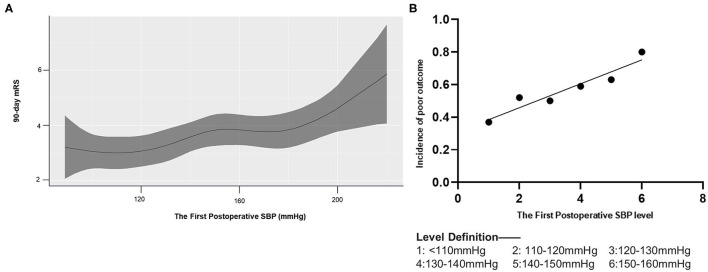
Association of 90-day mRS and the first postoperative SBP. mRS, modified Rankin Scale. **(A)** A generalized additive model with the line shows predictive values of 90-day mRS score according to the first postoperative SBP (the shade represents 95% CI of them). **(B)** Linear regression model for the first postoperative SBP and the incidence of poor outcome (mRS 3–6).

A generalized additive model showed that an increase in the first postoperative measurement of systolic blood pressure was associated with poor functional outcomes (Figure 2A). In fact, each 10-mmHg increase in this measurement, as long as it remained below 160 mmHg, raised the risk of poor outcome in a linear fashion (β = 0.073, *p* = 0.004, R^2^ = 0.91; Figure 2B). The Youden index identified a value of 136 mmHg as able to discriminate between patients who experienced good or poor functional outcomes. The area under the receiver operating characteristic curve was 0.63 (95%CI 0.54–0.71, *p* = 0.006), sensitivity was 57%, and specificity was 67%.

**Figure 3:**
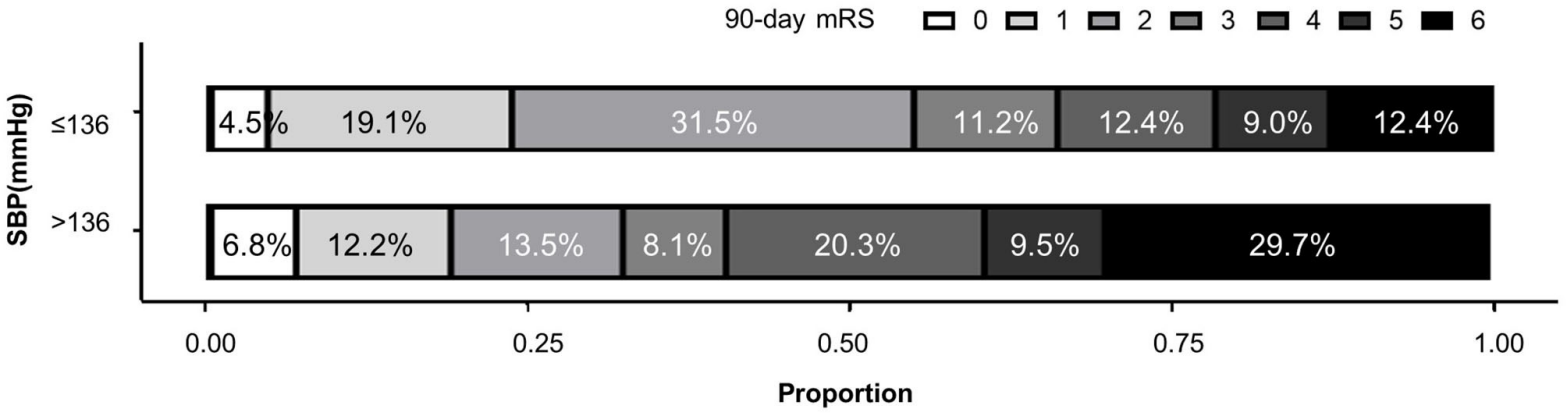
Distributions of 90-day modified Rankin Scale (mRS) in patients with different first postoperative SBP values.

Significant correlation of higher FPO BP dichotomized at 136 mmHg and poor functional outcome was shown in both univariate analysis (*p* = 0.004; OR 2.55; 95%CI 1.34–4.85) and logistic regression (model 1 *p* = 0.009, OR 0.395; 95%CI 0.20–0.79; model 2 *p* = 0.329, OR 0.69; 95%CI 0.32–1.46) (Figure 3). Patients with first postoperative systolic blood pressure above 136 mmHg had significantly higher postoperative blood pressure and worse quality of blood pressure management during the first 6 h after EVT (Supplementary Table 3). Their systolic blood pressure also took significantly longer to fall from the first postoperative level to the minimum level during the first 6 h.

### Associations of Intracerebral Hemorrhage and Baseline Characteristics or Various Blood Pressure Parameters During the First 24 h After EVT

Of the 163 patients we included, 61 patients (37%) had ICH during hospitalization. These patients were more likely to suffer malignant cerebral edema and have lower ASPECTS, higher NIHSS score and higher fasting blood glucose than patients without ICH (Supplementary Table 4). As data showed in Supplementary Table 5, no significant association was found between blood pressure parameters and intracerebral hemorrhage.

## Discussion

Our analysis of acute ischemic stroke patients who underwent EVT found that higher blood pressure within 6 h after the procedure correlated with worse functional outcomes at 90 days, particularly in patients with ICA occlusion or without cardiac dysfunction. In particular, using various approaches, we demonstrated an association between blood pressure within 30–40 min after EVT and functional outcome. Increasing blood pressure at this early time point correlated with increasing mRS score at 90 days, and blood pressure of 136 mmHg at this time point predicted reasonably well which patients later experienced poor or good functional outcome. Our study suggests that blood pressure should be adequately managed early after EVT to ensure good outcomes.

Differences in blood pressure parameters, especially postoperative blood pressure, between patients with good or poor functional outcomes were larger during the first 6 h after EVT than during other time windows within the first 24 h. Our results are consistent with previous studies reporting that clinical outcomes were significantly associated with mean blood pressure (12) or maximum systolic blood pressure (13) during the first 6 h after EVT. The importance of adequate blood pressure immediately after EVT may have several explanations. One is that cerebral autoregulation may be injured during the acute phase of ischemic stroke, leading to dysregulation of blood pressure (5, 14, 15). The effects of such dysregulation may be exacerbated by the large fluctuations in perfusion soon after recanalization. A second explanation may be that blood pressure soon after EVT may rise above preoperative levels but then decrease over the next several hours until stabilizing (6, 7). Blood pressure fluctuations may be more damaging when blood pressure is already high, which may help explain why functional outcomes can be so sensitive to blood pressure management early after EVT. Third, our previous study revealed that the extent of cerebral hyperdensities after EVT on postinterventional non-contrast-enhanced CT correlated with malignant brain edema (16). Whether early blood pressure parameters after EVT had an impact on postinterventional cerebral hyperdensities need more evidence.

To the best of our knowledge, this is the first study to report a potential association between blood pressure at 30–40 min after EVT and clinical outcomes. When patients were stratified according to whether systolic blood pressure at this time point was above or below 130 or 140 mmHg, no other blood pressure parameters showed significant associations with functional outcome. This implies that the various correlations of other blood pressure parameters with functional outcome reflect the influence of systolic blood pressure at 30–40 min. Consistent with this idea, we found that pressures at this time point that was below 160 mm were linearly associated with the risk of poor functional outcome. In our sample, a pressure of 136 mmHg predicted well whether our patients experienced good or poor outcomes at 90 days.

In our study, patients with higher systolic blood pressure at the first postoperative measurement had higher mean, minimum and maximum blood pressures, and they were at higher risk of poor mRS score at 90 days. This is consistent with previous reports that higher blood pressure can lead to poor functional outcomes in acute ischemic stroke patients (6, 9–11, 17, 18). These observations lead us to recommend strictly controlling blood pressure during the first 6 h or at least the first 24 h after EVT based on the first postoperative measurement of blood pressure.

Our subgroup analyses revealed that blood pressure parameters correlated significantly with functional outcomes in patients with ICA occlusion, but not in those without such occlusion. We originally thought that this result might reflect that ICA occlusion correlates with higher blood pressure at admission, but we did not detect such a correlation when we compared blood pressure between subgroups with or without occlusion. Therefore, other mechanisms may be responsible. One possibility is that ICA occlusion is associated with poor collateral circulation (19). Poor collateral circulation may compromise the body's ability to compensate for ICA occlusion, rendering tissues and vessels more sensitive to changes in perfusion.

## Limitation

Given the retrospective design of our study, we didn't collect blood pressure data 30–40 mins after EVT, which made it difficult to analyze and demonstrate the accuracy of the blood pressure profile at the early stage. Besides, we did we have data on NIHSS after EVT, so we could not explore the potential influence of symptomatic intracerebral hemorrhage on functional outcomes. All our patients underwent EVT while under general anesthesia, so our results may not be generalizable to those who undergo the procedure with local anesthesia, with or without conscious sedation. The type of anesthesia is known to affect blood pressure (20–22).

## Conclusion

Blood pressure of acute ischemic stroke patients during the first 6 h after EVT can significantly influence functional outcomes at 90 days. In fact, blood pressure at 30–40 min after the procedure may be independently associated with outcomes, highlighting the need to carefully manage blood pressure early after EVT.

## Data Availability Statement

The raw data supporting the conclusions of this article will be made available by the authors, without undue reservation.

## Ethics Statement

The studies involving human participants were reviewed and approved by Ethics Committee on Biomedical Research, West China Hospital of Sichuan University. Written informed consent for participation was not required for this study in accordance with the national legislation and the institutional requirements.

## Author Contributions

XZ: design of the study, methodology, data analysis, and article writing. TC: data collection and trimming. QZ: literature search and data collection. CW: data analysis direction. AW, YY, and SL: data collection. FH: direction of the study. BW: direction of the study and funding. All authors contributed to the article and approved the submitted version.

## Funding

This study was supported by National Natural Science Foundation of China (82071320 and 81870937) and 1.3.5 Project for Disciplines of Excellence, West China Hospital, Sichuan University (No. ZYGD18009).

## Conflict of Interest

The authors declare that the research was conducted in the absence of any commercial or financial relationships that could be construed as a potential conflict of interest.

## Publisher's Note

All claims expressed in this article are solely those of the authors and do not necessarily represent those of their affiliated organizations, or those of the publisher, the editors and the reviewers. Any product that may be evaluated in this article, or claim that may be made by its manufacturer, is not guaranteed or endorsed by the publisher.
